# Patellar retraction versus eversion on functional outcomes in total knee replacement: a randomized controlled study protocol

**DOI:** 10.1186/s13018-021-02518-y

**Published:** 2021-06-14

**Authors:** Zhao Wang, Yong Ji, Hongwei Bao, Jingzhao Hou, Yan-xiao Cheng

**Affiliations:** 1 Department of Orthopaedics, Jingjiang People’s Hospital, No. 28, Zhongzhou Road, Jingjiang, Taizhou City, 214500 Jiangsu Province China; 2 Department of General Surgery, Jingjiang People’s Hospital, No. 28, Zhongzhou Road, Jingjiang, Taizhou City, 214500 Jiangsu Province China

**Keywords:** Patellar eversion, Total knee replacement, Protocol

## Abstract

**Background:**

Patellar mobilization technique during total knee replacement (TKR) has been debated, with some suggesting that lateral retraction, rather than eversion, of the patella may be beneficial. This randomized controlled trial was to investigate the effects of patellar eversion on functional outcomes in TKR.

**Methods and analysis:**

This single-center, prospective, randomized controlled test will be conducted in Jingjiang People's Hospital. Primary end-stage osteoarthritis patients that prepared for unilateral TKR were randomized to one of two patellar exposure techniques during the primary total knee arthroplasty: lateral retraction or eversion. The informed consent will be acquired in each patient. The primary outcome was operation time, length of hospital stay, and straight leg raising time. Second outcomes including Insall-Salvati ratio; range of motion at 1 month, 3 months, and 1 year following TKR; visual analog scale (VAS) at 1 month, 3 months; and Knee Society Score (KSS) score at 1 year following TKR. The significance level was defaulted as P < .05.

**Results:**

Results will be published in relevant peer-reviewed journals.

**Conclusion:**

Our study aims to systematically assess the functional outcomes of patellar eversion for TKR patients, which will provide clinical guidance for TKR patients.

## Introduction

Total knee replacement (TKR) is among the most common elective procedures performed worldwide [1, 2]. However, up to 20% of patients are dissatisfied or not completely satisfied with the outcome of TKR [3]. In order to make patients more satisfied with the TKR, minimally invasive TKR was introduced and widely applied in the TKR surgeries [4]. It was reported that minimally invasive TKR obtained less blood loss, less loosening of lateral soft tissues, shorter hospital stay, and faster recovery of quadriceps [5].

The patella everted can augment surgical exposure and has been a routine part of surgical technique [6]. Lateral retraction of the patella is an alternative technique for TKR [7]. There is ongoing debate regarding whether or not the patella should be everted during TKR [8]. Zan et al. [9] conducted a meta-analysis and found that no difference in clinical outcome between patella eversion and lateral retraction in TKR. Jenkins et al. [10] found that lateral retraction of the patella did not lead to superior postoperative results compared with eversion of the patella during TKR. Reid et al. [11] found that retracting rather than everting the patella during TKR resulted in no significant clinical benefit in the early to medium-term follow-up. In current literature, there are limited randomized studies that specifically evaluate the results of patellar eversion versus patellar retraction during the TKR.

We thus designed a prospective, randomized controlled trial of 2 groups of patients treated with patellar eversion versus patellar retraction for TKR patients. Our goal was to compare functional outcomes at short-term and long-term follow-up between the two groups and finally provide clinical guidance for TKR patients.

## Methods and analysis

This study was registered in Research Registry (No: researchregistry6814).

### Participants

Patients with these conditions will be included (1) end-stage osteoarthritis patients; (2) primary unilateral TKR patients; (3) varus deformity or valgus deformity < 20°; and (4) the follow-up time was set to be at least 1 year after the surgical procedure. Exclusion criteria include (1) revision TKR; (2) previous knee surgery; (3) body mass index (BMI) ≥ 40.0 kg/m^2^; and (4) history of cardiovascular disease inability to tolerate the TKR procedure.

### Study design

This single-center, prospective, randomized controlled test will be conducted in Jingjiang People's Hospital. The sample size was based on the ability to detect a clinically important difference in postoperative quadriceps strength between groups. The protocol was approved by ethics committee of Jingjiang People's Hospital (JJHS-2019-527). Written informed consent will be obtained from each participating patient.

### Randomization and blind

Once the patients have read and signed the informed consent, those who have met the inclusion criteria will be randomly assigned to one of the two intervention groups: patellar eversion group (n = 50) and patellar retraction group (n = 50).

**Fig. 1:**
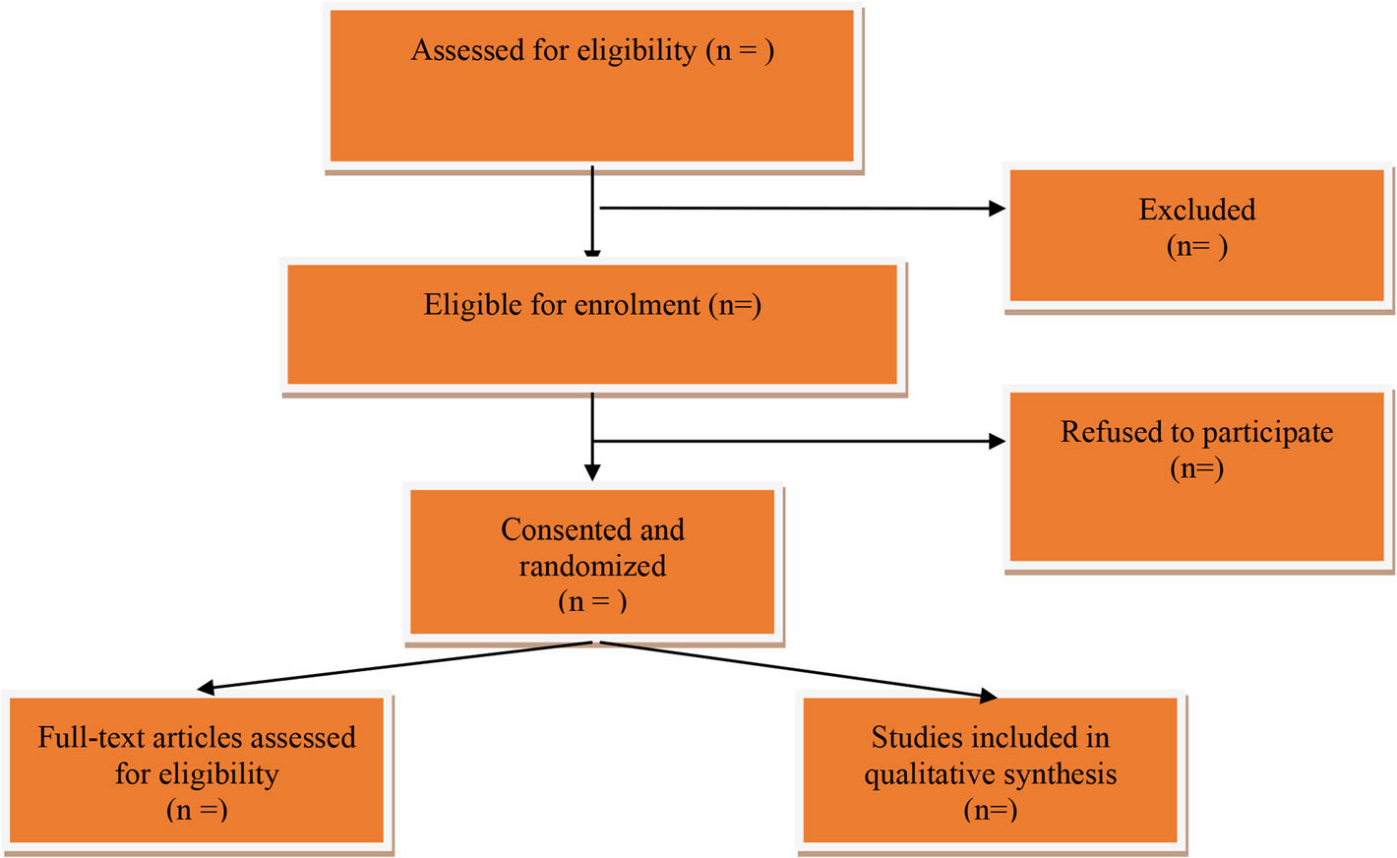
The flow diagram of a procedure to select studies

A computer-generated, permuted-block randomization scheme with a 1:1 ratio between the intervention and control groups will be used, and the software program Stata 14 (Stata Corp LLC, College Station, TX, USA) will be used to carry out the randomization. The allocation will be concealed using sealed and opaque envelopes, numbered consecutively. An independent researcher who will not participate in other study procedures will perform the randomization process. The flow diagram of the study is summarized in Fig. 1.

### Outcome assessment

Short-term outcomes were as follows: operation time, length of hospital stay, and straight leg raising time. Long-term clinical effect mainly including Insall-Salvati ratio; range of motion at 1 month, 3 months, and 1 year following TKR; and visual analog scale (VAS) at 1 month, 3 months, and Knee Society Score (KSS) score [12, 13] at 1 year following TKR. The ISR Insall-Salvati ratio is the ratio between the length of the patellar tendon and length of the patella. Visual analog scale (0-100 mm): The patients were asked to mark a standardised 100 mm visual analog scale (VAS) ranging from 0 to 100.

### Data analysis

All statistics were done using SPSS 21.0 (IBM SPSS Statistics for Windows, Version 21.0, Chicago, IL, USA) software. Results for normally distributed continuous variables are expressed as the mean value ± standard deviation, and continuous variables with nonnormal distribution are presented as median values and interquartile range. The statistical analysis was done using a two-tailed Student’s t test. P < 0.05 is considered statistically significant.

## Discussion

As a mature surgical technique for the treatment of severe osteoarthritis, TKR can relieve knee joint pain and correct knee joint deformity, improve knee joint function, improve the quality of life of patients, and the clinical effect is promising [14, 15]. In TKR surgery, it is necessary to move the patella to expose the surgical field of view [16]. In conventional TKR, the patella flip is often used to expose the surgical field of view, and the way the patella shifts laterally is more supported by researchers keen on minimally invasive surgery of TKR [17]. Theoretically, the advantage of lateral retraction of the patella is that it can reduce the pressure of the surgical operation on the knee extension device and reduce related soft tissue damage [11]. This procedure is more conducive to the recovery of postoperative extensor function.

The purpose of this randomized controlled trial was to conclude the effect of lateral retraction or eversion on the short-term and long-term functional outcomes of TKR. This study has some highlights. First, this is the first randomized controlled trial about the lateral retraction or eversion for TKR patients for long-term follow-up. In addition, we comprehensively assess the short-term and long-term functional outcomes of TKR. Moreover, we performed rigorous statistical analysis to increase the reliability of our study. Finally, we could provide evidence for clinical guidance.

## Data Availability

All the data pertaining to the present study are willing to share upon reasonable request.

## Abbreviations

TKR: Total knee replacement
BMI: Body mass index
VAS: Visual analog scale
KSS: Knee Society Score
